# Cytotoxicity of ammonia‐ and water‐based silver fluoride treatments

**DOI:** 10.1111/eos.70055

**Published:** 2025-11-26

**Authors:** Merve Uctasli, Roda Seseogullari‐Dirihan, Mustafa Murat Mutluay, Arzu Tezvergil‐Mutluay

**Affiliations:** ^1^ Department of Restorative Dentistry and Cariology Adhesive Dentistry Research Group Institute of Dentistry University of Turku Turku Finland; ^2^ Department of General Dental Science School of Dentistry Marquette University Milwaukee Wisconsin USA; ^3^ Department of Oral and Maxillofacial Diseases Faculty of Medicine University of Helsinki Helsinki Finland; ^4^ Turku University Hospital TYKS University of Turku Turku Finland

**Keywords:** aqueous silver fluoride, biocompatibility, dentin, potassium iodide, silver diamine fluoride

## Abstract

This study aims to investigate the trans‐dentinal and direct cell viability of ammonia‐ and water‐based silver fluoride treatments. Deep dentin discs were prepared, balanced for permeability and autoclaved. Three‐dimensional cultures of odontoblast‐like cells were transferred to the pulpal aspect of the dentin slices inside perfusion split chambers designed for dentin‐barrier cytotoxicity test, following ISO 7405. An experimental resin‐based glass ionomer cement and a polyvinylsiloxane impression material served as positive and negative controls, respectively. The experimental treatments included: (i) ammonia‐based silver fluoride = SDF, (ii) SDF + potassium iodide = KI, (iii) water‐based silver fluoride = SF, and (iv) SF + KI. Treatments were applied to the occlusal surface of dentin discs and cell viability (%) was assessed after 24 h using the methylthiazolium (MTT) assay. The cytotoxicity of dilutions (10^−3^, 10^−4^, and 10^−5^) were evaluated with direct exposure, using the same cell line following ISO 10993‐5. SF treatment revealed the highest cell viability among the treatment groups for the dentin‐barrier test. In direct cytotoxicity test, SDF and SF treatments exhibited no cytotoxicity at 10^−4^ and 10^−5^ dilutions. The addition of KI increased cytotoxicity. Ammonia‐ and water‐based silver fluoride treatments, particularly in deep cavities, should be applied with caution.

## INTRODUCTION

Dentin caries is a multifactorial and dynamic disease, recognized as the most prevalent oral health condition worldwide, affecting 2.5 billion people [1]. It continues to pose a major public health challenge [2]. This alarming prevalence highlights the urgent need for effective caries management strategies and the prioritization of oral health policies. Without timely and effective intervention, the growing burden of untreated caries will have a profound impact on healthcare systems in the coming decades.

The contemporary philosophy of caries management has shifted from the traditional drill‐and‐fill approach to a healing‐oriented model emphasizing preventive biological strategies that focus on preserving tooth structure and vitality [3]. Among the materials increasingly used in noninvasive or minimally invasive caries management, silver fluoride compounds have demonstrated remarkable efficacy in arresting caries progression and stand out as a promising option [4]. Silver fluoride treatments have two different chemical formulas depending on the third ion included. When silver and fluoride ions form a complex with ammonia, ammonia‐based silver fluoride, known as silver diamine fluoride (SDF), is formed [5]. Newer water‐based silver fluoride, in other words, aqueous silver fluoride (SF = AgF) composition has been marketed as an ammonia‐free alternative to the SDF solution. In this formulation, the ammonia ion is replaced by water and provides patient benefits such as improved smell, taste, and reduced soft tissue irritation. A well‐documented side effect of silver fluoride treatments that limits their use in clinical practice, is the characteristic silver staining of teeth and surrounding tissues due to oxidation of free silver ions [6]. However, this drawback has not significantly impacted the acceptance of silver fluoride treatments, especially in nonesthetic zones, among older adults, children, and high caries risk patients [7, 8, 9]. To address the discoloration issue, application of a generous amount of potassium iodide (KI) immediately after ammonia‐ and water‐based silver fluoride treatments promotes the formation of a white silver iodide compound that ameliorates the black dentin stains [6]. Therefore, silver fluoride treatments can be achieved either by ammonia‐ or water‐based silver fluoride alone (SDF or SF) or in combination with potassium iodide (KI).

The World Health Organization (WHO) Global Strategy on Oral Health [10] aims for 50% of countries to include SDF in their essential medicines list by 2030, emphasizing the growing importance and anticipated increased use of silver fluoride treatments in clinical practice. Despite their well‐documented clinical effectiveness, concerns remain regarding the cytotoxicity of both ammonia‐ and water‐based silver fluoride treatments, as silver and fluoride ions can cause toxic effects on human cells even at low concentrations [11]. Furthermore, studies have reported that silver fluoride treatments may induce mild chronic inflammatory responses in pulp tissues due to the deep penetration of silver particles into the dentin structure, particularly in teeth with deep caries lesions [12, 13].

While several studies have evaluated the cytotoxicity of ammonia‐based silver fluoride (SDF) [14, 15, 16], relatively few have investigated its combination with KI using different test models and cell lines [17, 18]. To date, only two studies have investigated the cytotoxic effects of diluted ammonia‐ and water‐based silver fluoride solutions on buccal mucosa fibroblasts [19] and dental pulp stem cells [20]. Current in vitro models for cytotoxicity assessment often involve exposing cells directly to diluted solutions or eluates of the materials, which neglects the potential protective role of dentin as a natural barrier between the material and the pulp. This highlights a critical gap in the literature regarding the lack of studies evaluating the trans‐dentinal cytotoxicity of ammonia‐ and water‐based silver fluoride treatments, where the material first interacts with dentin before ion diffusion toward pulp cells, more accurately mimicking the in vivo scenario [21].

Consequently, this study aimed to evaluate the effects of ammonia‐ and water‐based silver fluoride treatments on (i) trans‐dentinal cell viability in simulated deep clinical cavities and (ii) direct cell viability through dilutions. The null hypotheses tested were that ammonia‐ and water‐based silver fluoride treatments would have no effect on trans‐dentinal cytotoxicity or direct cell viability.

## MATERIAL AND METHODS

Sound third molars extracted during routine dental treatments from anonymous donors were used in the study and exempt from ethical notification in accordance with local regulations (Tissue act, section 20). Teeth were cleaned and stored in 0.9% NaCl supplemented with 0.02% NaN_3_ at 4°C to prevent bacterial growth and used within 3 months of extraction.

### Preparation of dentin disc specimens and measurement of dentin permeability

Preparation of dentin discs and measurement of dentin permeability were carried out according to ISO 7405 [22]. One hundred eighty teeth were sectioned perpendicularly to their long axis directly above the pulp horns under water‐cooling using a low speed diamond saw (Isomet 1000 Precision Saw, Buehler) to obtain one dentin disc from the deep dentin region of each tooth with the thickness of 0.5 mm and diameter of 10 mm. Absence of pulp horns and perforations were verified with a stereo microscope (Leica M60, Leica Microsystems) at 40× magnification. Dentin discs indicating perforations or pulpal exposures were discarded and replaced. Then, dentin discs were polished from the occlusal surfaces with 320‐grit silicon carbide (SiC) paper (CarbiMet, Buehler) to obtain dentin discs with a final thickness of 0.4 (±0.02) mm. The occlusal surface of each dentin disc was determined based on the conical geometry of dentinal tubules. The occlusal surface of each dentin disc was marked. The thickness and diameter of each disc were measured by a digital micrometer (Mitutoyo).

Prepared dentin disc surfaces were etched for 30 s with 50% citric acid and rinsed for 30 s with distilled water to remove the smear later. Dentin permeability was evaluated through a flow‐measurement infiltration apparatus (SLI‐1000 Liquid Flow Meter, Sensirion) in a modified split‐chamber unit, which was linked to a deionized water container at a simulated hydrostatic pressure of 20 cm [23]. Dentin discs (*n* = 30 dentin disc/treatment group) were allocated homogeneously into groups depending on their individual permeability values. Eight dentin discs with similar permeability values were used in each dentin barrier cytotoxicity test setup. After permeability measurements, dentin discs were rinsed with distilled water and autoclaved in 0.9% sodium chloride (NaCl) at 121°C for 25 min.

### Preparation of pulp‐derived three‐dimensional cell culture

The immortalized clonal large T‐antigen transfected bovine pulp‐derived cells (SV40) derived from calf dental papilla [22, 24], of which we received the clonal subline “SVNeo3B cells” [24], were provided as a kind donation from Regensburg University and stored frozen in liquid nitrogen until use. Cells were passaged and maintained in growth medium (Alpha Modification of Eagle's Minimum Essential Medium (α MEM) M8042, Merck Sigma‐Aldrich) supplemented with 20% fetal bovine serum (FBS) (Gibco, Thermo Fisher), 150 IU/mL penicillin, 150 mg/mL streptomycin, 0.125 mg/mL amphotericin B, and 0.1 mg/mL geneticin (Merck Sigma‐Aldrich) at 37°C, 5% CO_2_, and 100% humidity [22]. For all the experiments, cells within passages 19–24 were used [25]. Polyamide nylon meshes (pore size 150 µm, diameter 8 mm) were cleaned with 0.1 M acetic acid for 30 min at room temperature, rinsed three times with distilled water and coated with 0.03 mg/mL fibronectin (bovine plasma fibronectin, Merck Sigma‐Aldrich). A six‐well tissue culture plate was filled with 1.25 mL of α MEM supplemented with 20% FBS. Millicel inserts (Merck Sigma‐Aldrich) were placed at the bottom of each well on the well plate to support the initial growth of the cells on the polyamide nylon meshes. Four polyamide nylon meshes were inserted into each insert. Calculations of cells on polyamide nylon meshes were done with TC20 automated cell counter (Luminex xMAP, BioRad) by mixing 15 µL of 0.4% trypan blue and then pipetting 10 µL of the mixture into the counting slide chamber. Cell suspensions were adjusted to 80.000 cells/20 µL for each polyamide nylon mesh and the cultures were incubated for 48 h to allow proper cell growth at 37°C, 5% CO_2_, and 100% humidity. After incubation, polyamide nylon meshes were transferred to a 24‐well plate and were separately placed inside the wells. In each well, 1 mL of α MEM and 20% FBS medium were added to feed cells. The medium was changed three times a week for 14 days to produce cells on the polyamide nylon meshes in a three‐dimensional form. Extra polyamide nylon meshes were prepared for each test with an adequate number of cells on the membranes. To choose the eight polyamide nylon meshes with three‐dimensional cultures of pulp‐derived cells exhibiting similar cell viabilities, the cells were transferred to a new 24‐well plate on the day of the experiment. Each well contained 400 µL α MEM and 20 µL cell proliferation reagent (WST‐1, Roche). After 1 h of incubation, the optical density was measured at 440 nm using a spectrophotometer (Synergy HT, BioTek Instruments). Meshes with similar cell viabilities (*n* = 8) were selected for use in each dentin barrier test setup.

### Dentin barrier cytotoxicity test (trans‐dentinal cytotoxicity)

The dentin barrier test method was carried out according to ISO 7405 [22]. In this method, tested materials are applied over dentin discs and allowed to pass through dentinal tubules to evaluate cell response inside split‐chamber compartments [26]. After 14 days of incubation and cell viability measurements, polyamide nylon meshes containing three‐dimensional cell cultures were placed in the lower compartment of commercially available individual perfusion split chambers (Minucells and Minutissue, Bad Abbach) in direct contact with the pulpal side of the prepared dentin disc and held in place by a stainless‐steel holder. The pulpal compartment was perfused with assay medium supplemented with 5.96 g/L hydroxyethylpiperazine ethane sulfonic acid (HEPES) at a rate of 0.3 mL/h for 24 h using a precision pump (Minucells and Minutissue, Bad Abbach). Perfusion was then briefly halted, and the surfaces were gently rinsed with distilled water for 30 s. Ammonia‐ and water‐based silver fluoride treatments, SDF and SF, were applied for 60 s and for the groups including the second step KI treatment, KI were applied immediately after SDF and SF for 60 s until the creamy white appearance turned clear. All treatments were applied with a microbrush into the upper chamber in direct contact with the occlusal surface of the dentin disc and dried. The tested silver fluoride treatment groups were (i) ammonia‐based silver fluoride = silver diamine fluoride = SDF (Riva Star Bottle 1, SDI); (ii) SDF + potassium iodide = KI (Riva Star Bottle 1 + 2, SDI); (iii) water‐based silver fluoride = aqueous silver fluoride = SF (Riva Star Aqua Bottle 1, SDI); (iv) SF + KI (Riva Star Aqua Bottle 1 + 2, SDI). According to ISO 7405 B.3 [22], an experimental resin‐based glass ionomer cement [27], which is known to cause toxic effects, was prepared and served as the positive control (50% cell viability), while a polyvinylsiloxane impression material (Express VPS, Solventum) was used as the negative control (100% cell viability).

After closing the split chambers, cells were perfused at a rate of 0.2 mL/h for 24 h. Cell meshes were gently sectioned by the metallic inserts into 4 mm^2^ circular pieces, retrieved from the stainless‐steel holder and placed into 24‐well plates containing 0.5 mL of methylthiazolium (MTT) solution. The 24‐well plates were incubated for 2 h at 37°C, 5% CO_2_, and 100% humidity. Following incubation, polyamide nylon meshes were washed with phosphate buffered saline and transferred to a 48‐well plate containing 0.25 mL dimethyl sulfoxide. The blue formazan precipitate was extracted from the mitochondria using 0.25 mL dimethyl sulfoxide on a shaker at room temperature for 15 min. About 200 µL of this solution were transferred to a 96‐well plate. Cell viability were assessed by the MTT assay and read by a spectrophotometer (Synergy HT, BioTek Instruments) at 540 nm.

In each dentin barrier test setup, eight dentin discs with similar permeabilities and eight polyamide nylon meshes with similar cell viability were used. Each experiment consisted of eight perfusion split chambers and the dentin barrier test was repeated until 30 readings were obtained for each material tested. Positive and negative controls were included in each test, and all components of the test setup were sterilized after each experiment. The mean absorption values of the negative and positive controls were set to represent 100% and 50% cell viability, respectively. Results were calculated as percentages based on the plotted curve derived from the negative and positive control samples.

### Direct cell viability assay to evaluate the cytotoxicity of dilutions

Direct cell viability assay test was conducted according to ISO 10993‐5 [28]. The same cell line (SV40) were used to test the effect of dilutions of ammonia‐ and water‐based silver fluoride treatments. Cells were seeded inside a 96‐well plate at a density of 1 × 10^4^ cells per well and surrounded by medium. After 24 h incubation in a humified atmosphere at 37°C, 5% CO_2_, and 100% humidity, the media was replaced and cells were exposed to the dilutions (100 µL). Three different dilutions as 10^−3^, 10^−4^, or 10^−5^ were prepared in cell media for each ammonia and water‐based silver fluoride treatment group: (i) SDF, (ii) SDF + KI, (iii) SF, and (iv) SF + KI. Based on manufacturer specifications, the SDF solution contained 38% w/v silver fluoride, 15%–20% w/v ammonia and 40%–60% w/v water. The SF solution also contained approximately 38% w/v silver fluoride and 50%–70% w/v water. The KI solutions contained 58% w/w potassium iodide. For the SDF and SF groups, the respective stock solutions were directly diluted in cell media at 10^−3^, 10^−4^, or 10^−5^ dilutions. For the SDF + KI and SF + KI groups, equal volumes of the either ammonia‐ or water‐based silver fluoride solution and KI were mixed and then the resulting mixture was diluted in cell media to obtain final concentrations. The mean pH values of dilutions were determined by a digital pH meter (Radiometer Analytical). Fresh media exposure served as negative control. To test the effect of dilutions after 24 h of incubation, plates were examined under microscope for morphological alterations. Then, culture medium was removed from each well and 50 µL of 0.5 mg/mL MTT solution was added and incubated at 37°C, 5% CO_2_, and 100% humidity. After 3 h, MTT solution was removed and 100 µL of dimethyl sulfoxide was added to each well. The optical density was quantified using a spectrophotometer (Synergy HT, BioTek Instruments) at 570 nm. Three independent experiments were performed in triplicate. The percent cell viability was calculated using the optical density of negative control samples set to 100%.

### Statistical analyses

Data were compared for normality (Shapiro–Wilk) and homoscedasticity (Levene). Since trans‐dentinal cytotoxicity dentin barrier test data were not normally distributed, data were analyzed by the Kruskal‐Wallis test followed by the Dunn's test. Assessment of cell damage was further categorized as noncytotoxic, moderately cytotoxic, and severely cytotoxic according to ISO 7405 [22]. Direct cell viability assay data were normally distributed and homoscedastic. Data were analyzed with one‐way ANOVA followed by the Tukey test. Significance level (α) of 0.05 was used for all statistical tests. Statistical analyses were performed using ibm spss statistics for windows, version 28 (IBM).

## RESULTS

### Dentin barrier cytotoxicity test (trans‐dentinal cytotoxicity) cell viability

**FIGURE 1:**
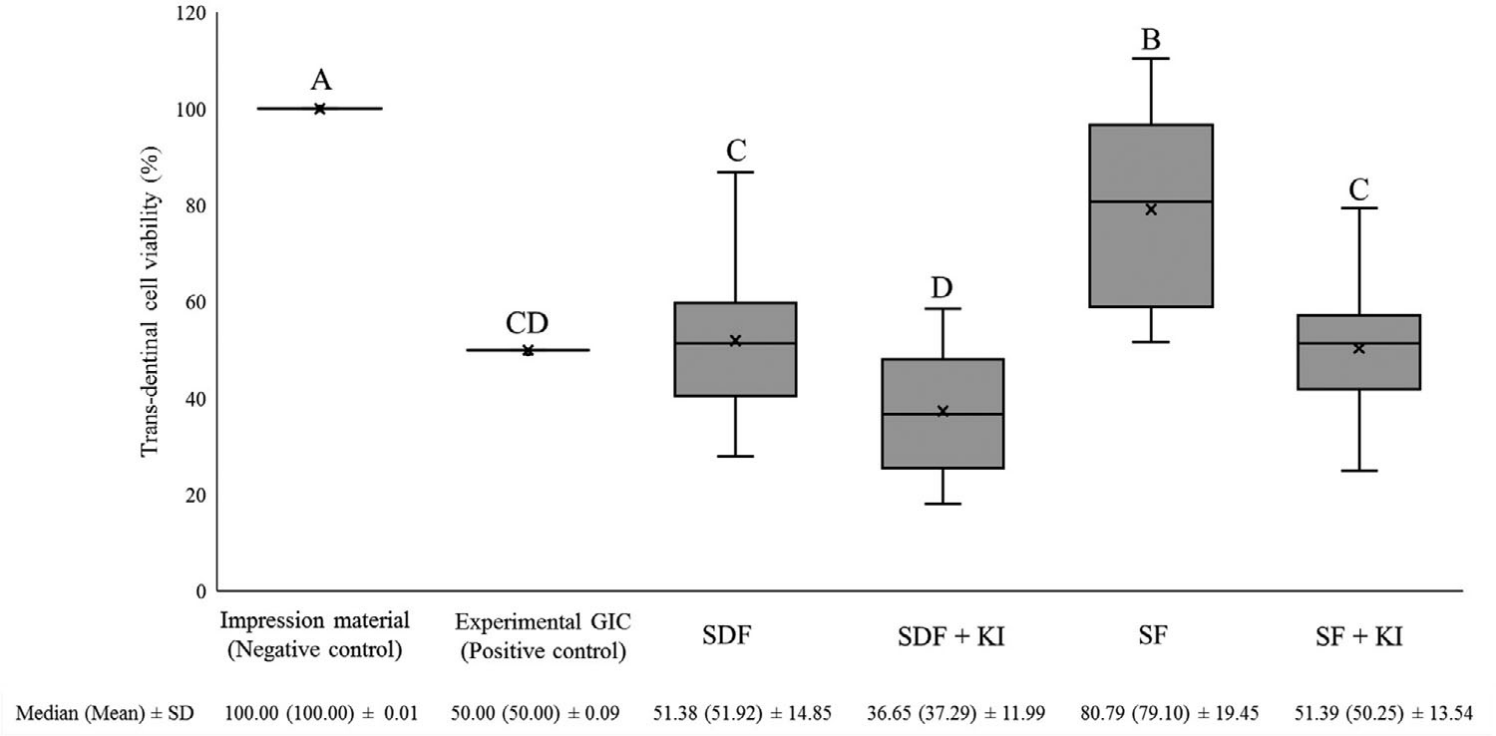
Box‐plot diagram representing the percentage trans‐dentinal cell viability (%) of SV40 pulp‐derived cells exposed to ammonia‐ and water‐based silver fluoride treatments (*n* = 30). The horizontal line inside each box represents the group median, while the “x” inside each box represents the group mean. Different upper‐case letters indicate statistically significant differences between the groups according to Dunn`s test (*p *< 0.05). The median, mean and standard deviation of all groups are given below the figure. GIC, glass ionomer cement; KI, potassium iodide; SD, standard deviation; SDF, silver diamine fluoride; SF, aqueous silver fluoride.

The percentage cell viability, along with the median, mean, and standard deviation values of ammonia‐ and water‐based silver fluoride treatments after 24 h are shown in Figure 1. Kruskal–Wallis test revealed that ammonia‐ and water‐based silver fluoride treatments significantly affected cell viability (*p *< 0.001). All treatment groups demonstrated significantly lower cell viability compared to the negative control (impression material) (100% cell viability), with SF showing approximately 20% lower viability, the positive control (experimental resin‐based glass ionomer cement), SDF and SF + KI showing around 50% lower viability and SDF + KI showing about 63% lower cell viability. Among the treatment groups, the water‐based silver fluoride treatment, SF, revealed the highest cell viability (79% cell viability). The addition of potassium iodide (KI) as the second step significantly reduced cell viability compared to their respective single‐step ammonia‐ and water‐ based treatments, SDF + KI (37% cell viability) was lower than that for SDF (52% cell viability), and the cell viability for SF + KI (50% cell viability) was lower than that observed for SF (79% cell viability). Notably, SDF + KI group exhibited the lowest cell viability compared to all other treatment groups. There were no statistically significant differences between the positive control (experimental resin‐based glass ionomer cement), SDF and SF + KI groups, indicating comparable cell viabilities.

### Dentin barrier cytotoxicity test (trans‐dentinal cytotoxicity) assessment of cell damage

Grading assessments of cell damage as noncytotoxic, moderate cytotoxic, and severe cytotoxic according to ISO 7405 [22] are presented in Table 1 and pairwise comparisons are shown in Figure 1. Statistically significant differences were observed between negative (impression material) and positive (experimental resin‐based glass ionomer cement) control groups (*p* < 0.001). According to ISO‐based cell damage grading [22], the water‐based silver fluoride treatment, SF, were classified as moderately cytotoxic, showed a statistically significant different from both the negative and positive control groups. All other treatment groups, SDF, SDF + KI, and SF + KI, were classified as severely cytotoxic, with cell viability values being statistically significantly lower than seen in the negative control group (*p *< 0.05), but not differing statistically significantly from the positive control group.

**TABLE 1:** Assessment of cell damage (SV40 pulp‐derived cells) according to ISO 7405 after ammonia‐ and water‐based silver fluoride treatments.

Material	Cell damage
Ammonia‐based silver fluoride	
SDF	Severely cytotoxic
SDF + KI	Severely cytotoxic
Water‐based silver fluoride	
SF	Moderately cytotoxic
SF + KI	Severely cytotoxic

Abbreviations: KI, potassium iodide; SDF, silver diamine fluoride; SF, aqueous silver fluoride.

### Direct cell viability assay cytotoxicity of dilutions

**FIGURE 2:**
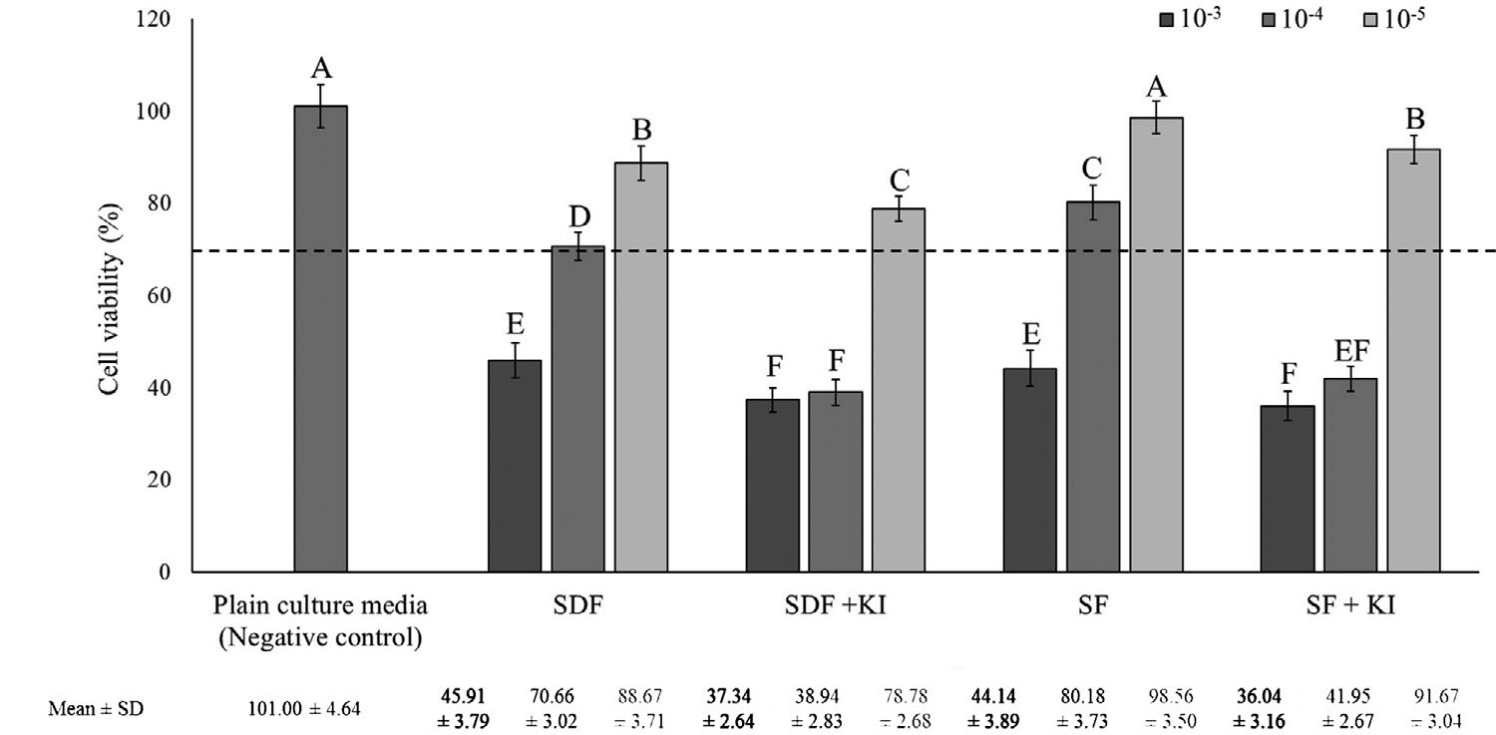
Cell viability (%) of SV40 pulp‐derived cells exposed to prepared dilutions (10^−3^, 10^−4^, and 10^−5^) of ammonia‐ and water‐based silver fluoride treatments. Fresh culture media served as negative control and set to 100% cell viability. The dashed line represents ISO 10993 cut‐off level (70%), bars below the dashed line represent cytotoxicity and bars above the dashed line represent noncytotoxicity. Different upper‐case letters indicate significant differences between the groups according to Tukey test (*p* < 0.05). KI, potassium iodide; SDF, silver diamine fluoride; SF, aqueous silver fluoride.

The percentage of viable cells according to the direct cell viability assay test for ammonia‐ and water‐based silver fluoride treatments are summarized in Figure 2. One‐way ANOVA revealed that ammonia‐ and water‐based silver fluoride treatments significantly affected the cell viability (*p* < 0.001). According to ISO 10993‐5 [28], 70% and higher cell viability means that the material is non‐cytotoxic as marked with a dotted line in Figure 2. At 10^−3^ dilutions, the ammonia‐ and water‐based silver fluoride treatments revealed cytotoxic effects with cell viabilities around 30% which is significantly lower than seen for the negative control (plain culture media) (*p* < 0.05). At 10^−4^ dilutions, cells exposed to SDF and SF revealed 70% or higher cell viability resulting in being noncytotoxic. SF treatment revealed higher cell viability compared to SDF (*p *< 0.05). The groups involved KI treatment revealed cell viabilities around 40%. All treatment groups revealed noncytotoxicity by passing the 70% cut‐off level at 10^−5^ dilutions. There were no significant differences between SF and negative control (*p* > 0.05).

The mean pH values of ammonia‐ and water‐based silver fluoride treatments are shown in Table 2. The ammonia‐based silver fluoride treatments revealed alkaline pH at dilutions less than 10^−5^, whereas water‐based silver fluoride treatments revealed neutral pH. Similar pH values were obtained at 10^−5^ dilution for both ammonia‐ and water‐based silver fluoride treatments. The additional KI treatment did not affect the pH values.

**TABLE 2:** Ph values mean (± standard deviation) of the diluted ammonia‐ and water‐based silver fluoride treatments.

Dilutions				
Material	10^−2^	10^−3^	10^−4^	10^−5^
Ammonia‐based silver fluoride				
SDF	9.47 (±0.06)	8.30 (±0.07)	8.05 (±0.06)	7.87 (±0.04)
SDF + KI	9.46 (±0.05)	8.30 (±0.05)	8.03 (±0.06)	7.85 (±0.05)
Water‐based silver fluoride				
SF	7.73 (±0.05)	7.78 (±0.08)	7.71 (±0.07)	7.70 (±0.07)
SF + KI	7.79 (±0.07)	7.73 (±0.05)	7.70 (±0.06)	7.69 (±0.05)

Abbreviations: KI, potassium iodide; SDF, silver diamine fluoride; SF, aqueous silver fluoride.

## DISCUSSION

Biocompatibility refers to the ability of a material to safely perform its intended therapeutic function without causing undesirable local or systemic effects, such as toxicity, inflammation, or immune rejection [29]. Traditionally, animal models have been employed to evaluate the biocompatibility of dental material with pulp; however, contemporary research increasingly relies on in vitro models, including cytotoxicity assays, protein adsorption, cell adhesion tests, tissue‐based studies, and dentinal barrier assessments using models such as the dentin barrier, in vitro pulp chamber, and tooth‐on‐chip [14, 16, 30].

In the present study, two in vitro cytotoxicity tests were conducted to assess the cytotoxicity of ammonia‐ and water‐based silver fluoride treatments. The first method involved applying the treatment to a dentinal barrier, which served as a biological barrier through which the applied treatments could diffuse to reach the cells. The second method directly exposed cells to the dilutions of the treatments to evaluate their direct cytotoxicity. Both tests were performed according to ISO standards [22, 28], allowing a comparison of results obtained from the two methods (indirect vs. direct contact). ISO‐standardized approach is crucial for the comparability of the data and the data quality. The tested ammonia‐ and water‐based silver fluoride treatments affected the trans‐dentinal viability of pulp‐derived cells and the dilutions of the same treatments influenced the direct cell viability assay, leading to the rejection of both null hypotheses.

Trans‐dentinal cytotoxicity tests provide a more clinically relevant assessment than dilution‐based direct cytotoxicity assays, given that dentin plays a critical protective role and significantly influences the compatibility of materials applied in deep cavities [21, 26]. Since dentin is a semi‐permeable structure with permeability variations across a few millimeters [31], dentin disc permeabilities were evaluated to ensure consistency in each test setup. Additionally, the controlled flow of culture media through a peristaltic pump simulated physiological blood flow, supplying fresh nutrients and washing away potentially toxic compounds [21]. Treatments were applied according to the manufacturer's clinical recommendations to ensure clinical relevance, for 60 s using a microbrush. Furthermore, baseline cell viability of polyamide nylon meshes were confirmed prior to testing to ensure uniform conditions.

Conversely, the dilution‐based direct cytotoxicity assay, which exposes cells to ammonia‐ and water‐based silver fluoride treatment dilutions, is less clinically relevant [30]. The stability of reagents in the presence of silver fluoride treatments was validated before testing and potential interference from pH changes or reactive components were carefully considered and managed (Table 2). The dilutions were chosen based on preliminary experiments which showed that the more concentrated 10^−2^ dilution of ammonia‐ and water‐based silver fluoride resulted in turbid media that could cause misleading results; therefore, 10^−2^ data were excluded, and dilutions of 10^−3^, 10^−4^, and 10^−5^ were used, aligning with previous studies [14, 15, 18]. In terms of clinical relevance, the concentrations tested in this study were selected to approximate potential pulpal exposures. In clinical practice, a single drop of either ammonia‐ or water‐ based silver fluoride solution typically used to treat up to five tooth surfaces. The actual amount of material reaching the dentin and potentially diffusing toward the pulp is therefore limited and subject to progressive dilution by dentinal fluids. The 10^−3^ dilution used in our experiment represents a maximum exposure immediately following application. The 10^−4^ and 10^−5^ dilutions simulate the gradual dilution and diffusion effects that would occur physiologically over time.

Both trans‐dentinal cytotoxicity and the dilution‐based direct cytotoxicity tests were evaluated using the MTT assay [22, 28], a rapid widely utilized assay that measures mitochondrial metabolic activity. MTT assay detects the reduction of the yellow tetrazolium salt (3‐(4,5‐dimethylthiazol‐2‐yl)‐2,5‐diphenyltetrazolium bromide) into purple formazan crystals and is analyzed spectrophotometrically [26, 30]. However, as the MTT assay relies on colorimetric changes rather than direct visualization of cells, certain materials can alter pH, and affect assay reliability, as observed in 10^−2^ dilutions (Table 2). This issue was minimized in the trans‐dentinal cytotoxicity test, where cell viability was assessed indirectly from polyamide nylon meshes using the MTT assay. Both tests incorporated strict controls and standard curves to ensure reliability [22, 28].

Cell line selection significantly influences toxicity assay results. The current study followed ISO standards [22] and used clonal large T‐antigen bovine pulp‐derived cells (SV40) [24, 28]. The immortalized clonal SV40 transfected bovine pulp‐derived cells, originally derived from calf dental papilla, have been widely used in three‐dimensional dentin barrier tests as a standardized and reproducible in vitro model [21, 25, 26]. Their ability to differentiate into odontoblast‐like cells supports their relevance as a proxy for pulp cells and the three‐dimensional culture system further enhances physiological relevance by allowing cell–cell and cell–matrix interactions and realistic diffusion of test substances [24, 30]. However, different oral tissues may exhibit unique responses, highlighting the need to assess cytotoxicity in various cell types relevant to clinical applications, such as pulp‐derived cells for deep cavities [15, 20] and gingival fibroblasts for cervical applications [14, 19].

The Riva Star and Riva Star Aqua (SDI) silver fluoride products were selected due to their widespread clinical use and commercial availability, especially in Europe, Asia, and Australia. The 38% silver fluoride concentration has been reported as the most effective concentration [32], which further supports our material selection. Other commercially available 38% silver fluoride treatments include Advantage Arrest (Elevate Oral Care) [14], Saforide (Tokyo Seiyoku Kasei), Bioride (Dentsply Industria e Comercio), e‐SDF (Kids‐e‐dental), Fagamin (NAF Laboratorios), and Topamine (Pharmadesign). The varying fluoride ion concentrations among these products make direct product comparisons unfeasible [33], and with the global market for silver fluoride treatments expanding, further research is required to evaluate the cytotoxicity of different commercially available products.

This in vitro study investigated the direct and indirect cytotoxic effect of ammonia‐ and water‐based silver fluoride treatments. To our knowledge, this is the first study to evaluate the cytotoxicity of a water‐based silver fluoride treatment using a trans‐dentinal cytotoxicity test. The findings of the present study indicate that water‐based silver fluoride exhibits lower cytotoxicity than ammonia‐based silver fluoride, aligning with previous direct cell viability study [20]. The dilution‐based direct cell viability test demonstrated a dose‐dependent cytotoxicity, consistent with previous studies [14, 15, 16, 20]. At 10^−5^ dilutions, all silver fluoride treatments passed the ISO‐defined noncytotoxicity cut‐off level at 70% cell viability [28], whereas at 10^−3^ dilutions, all treatments exhibited cytotoxicity.

Consistency observed between the results from indirect and direct cytotoxicity tests further supports our findings. Notably, water‐based silver fluoride (SF) without potassium iodide (KI) demonstrated the lowest cytotoxicity in both cytotoxicity tests. According to ISO 7405 standards [22], the results of trans‐dentinal cytotoxicity test were further evaluated to determine the cell damage. Based on cell damage assessment, a material is considered noncytotoxic when it is not statistically different from the negative control but differs significantly from the positive control. A material is classified moderately cytotoxic when it is statistically different from both the negative and the positive control. A material is considered severely cytotoxic when it is not statistically different from the positive control but significantly different from the negative control. SF exhibited moderate cytotoxicity, whereas ammonia‐based silver fluoride (SDF), SDF + KI, and SF + KI displayed severe cytotoxicity. Differences between ammonia‐ and water‐based silver fluoride treatments likely stem from the ammonia and water content in SDF and SF formulations, respectively. Previous studies indicate that fibroblasts exhibit reduced viability at pH values of 10 or higher [34] and the alkaline pH resulting from the ammonia ion in SDF has been associated with cell damage and reduced cell proliferation [35]. In contrast, the more neutral pH of SF allows for controlled diffusion through dentin in low doses, increasing the rate of secondary dentin formation rather than damaging the dentin‐pulp complex [12, 19]. Thus, water‐based SF may not only be a safer option but also more beneficial than ammonia‐based SDF [18, 20]. Additionally, differences in particle charge and size may affect cytotoxicity variations [30]. Positively charged ammonia and silver ions in SDF interact strongly with cell membranes, increasing cytotoxicity, whereas neutrally charged water ion in SF improves biocompatibility. Moreover, children who received ammonia‐based SDF on active dentin caries reported tooth pain as an adverse effect, further supporting SDF's cytotoxic potential [36].

The black stain caused by SDF and SF treatments remains a major clinical drawback, and the application of KI is recommended to minimize discoloration [6]. However, in this study, KI significantly increased cytotoxicity when applied as a second step after ammonia‐ and water‐based silver fluoride treatments. Interestingly, our findings contradict previous studies, which found the additional KI application noncytotoxic [17, 18, 20]. These discrepancies may arise from differences in cell lines, dilution ratios (1:2 in previous studies vs. 1:1 in our study) [18, 20] and dentin demineralization status [17]. Furthermore, scanning electron microscope analysis revealed that dentin surfaces were covered with spherical, small particles after ammonia‐ and water‐based silver fluoride treatments with an even greater accumulation observed following KI application [23]. Ions from these treatments have been shown to penetrate dentinal tubules, with penetration depth increasing linearly with tubule size [13]. Some studies have reported penetration depths of 1–1.5 mm [37] while others have detected particles deeper than 2 mm within dentinal tubules [13]. However, the effect of KI on penetration depth requires further investigation. The manufacturer reported KI concentration of 58% w/w and claimed identical formulations in Riva Star and Riva Star Aqua products. Our findings suggest that KI in Riva Star Aqua exhibits better biocompatibility, which is consistent with a previous study [19]. This difference could be related to the composition of the first step, the presence of water or ammonia and how these components interact with KI. The cytotoxic effect of KI with SDF and SF have not been fully described and further research is needed to clarify these mechanisms.

The current literature on ammonia‐ and water‐based silver fluoride treatments and their effects on dentin pulp tissue remains limited. Evaluating material biocompatibility is crucial to identify clinical limitations, ensure patient safety and facilitate regulatory approvals [30]. Future in vitro studies using different products, methodologies, cell lines, application durations, and frequencies should be conducted and their findings have to be validated with in vivo studies.

Although significant progress has been made in understanding the biocompatibility of dental materials, the results of in vitro cytotoxicity tests should not be directly extrapolated to clinical scenarios. The cytotoxic effects observed in this study emphasize the importance of selecting suitable silver fluoride treatments based on clinical circumstances. We can conclude that ammonia‐ and water‐based silver fluoride treatments should be applied with caution due to their demonstrated cytotoxic potential. Among the tested treatments, water‐based silver fluoride (SF), without the second step application of potassium iodide (KI) appears to be the most promising and safest option, particularly in cavitated lesions that may not be far from the pulp.

## AUTHOR CONTRIBUTIONS

**Conceptualization**: Uctasli M, Seseogullari‐Dirihan R, Mutluay MM, Tezvergil‐Mutluay A; **Investigation**: Uctasli M, Seseogullari‐Dirihan R; **Methodology**: Uctasli M, Seseogullari‐Dirihan R, Mutluay MM, Tezvergil‐Mutluay A; **Formal analysis**: Uctasli M, Mutluay MM; **Visualization**: Uctasli M; **Writing—original draft**: Uctasli M; **Writing—review and editing**: Uctasli M, Tezvergil‐Mutluay A; **Supervision**: Mutluay MM, Tezvergil‐Mutluay A; **Resources**: Tezvergil‐Mutluay A; **Funding acquisition**: Tezvergil‐Mutluay A.

## CONFLICT OF INTEREST STATEMENT

The authors declare no conflicts of interest.
